# Urine electrolytes guide treatment of Gitelman syndrome and recurrent syncope—A case report

**DOI:** 10.14814/phy2.71043

**Published:** 2026-09-21

**Authors:** Saloni Agrawal, Kenneth M. Fifer, Samira S. Farouk, Joshua L. Rein

**Affiliations:** ^1^ Department of Medicine Icahn School of Medicine at Mount Sinai New York New York USA; ^2^ Division of General Internal Medicine, Department of Medicine Icahn School of Medicine at Mount Sinai New York New York USA; ^3^ Barbara T. Murphy Division of Nephrology, Department of Medicine Icahn School of Medicine at Mount Sinai New York New York USA; ^4^ Renal Section, Department of Medicine James J. Peters Veterans Affairs Medical Center Bronx New York USA

**Keywords:** electrolytes, Gitelman syndrome, hypokalemia, kidney, potassium, syncope

## Abstract

Syncope is a common reason for inpatient admission. Few case studies describe an association between syncope and Gitelman syndrome (GS). Herein, we present a case of a woman with hypokalemia and recurrent syncope which eventually led to a diagnosis of GS. A physiologic approach to urine electrolyte interpretation informed clinical decisions to diagnose and manage complications associated with GS. Genetic testing for renal electrolyte transport‐related gene mutations among patients with recurrent syncope and electrolyte abnormalities may be diagnostic.

## 1 INTRODUCTION

Gitelman syndrome (GS) is an uncommon and incompletely understood, autosomal recessive, salt‐losing tubulopathy that was first described in 1966 (Gitelman et al., 1966). Inactivating mutations in *SLC12A3*, which encodes the thiazide‐sensitive sodium‐chloride cotransporter (NCC) cause GS (Simon et al., 1996). NCC is expressed exclusively at the apical membrane of epithelial cells in the kidney distal convoluted tubule (DCT) (Hoorn et al., 2020). Impaired conservation of sodium (Na^+^), potassium (K^+^), chloride (Cl^−^), and magnesium (Mg^2+^) leads to hypokalemic metabolic alkalosis with hypomagnesemia and normotensive secondary hyperreninism. GS is one of the more commonly inherited renal tubulopathies with an estimated prevalence of ~1–10 per 40,000 people (Blanchard et al., 2017). GS typically presents during adolescence or adulthood and has wide phenotypic variability associated with non‐specific symptoms such as muscle weakness or cramps, fatigue, salt craving, thirst, and polydipsia– less commonly with palpitations and cardiac arrhythmias attributed to electrolyte derangements that can cause syncope or cardiac arrest (Blanchard et al., 2017). However, although mechanistically plausible, few case studies describe the association between syncope and GS. Herein, we present a case of a woman with hypokalemia and recurrent syncope which eventually led to a diagnosis of GS. A solid understanding of renal tubular physiology and a pragmatic approach to urine electrolyte interpretation informed clinical decisions to diagnose and manage complications associated with GS. This case report is presented in accordance with CARE checklist guidelines (Supplemental File 1).

## 2 CASE REPORT

A 53‐year‐old woman with hypokalemia since at least her teenage years was directly admitted from outpatient primary care clinic for inpatient workup of recurrent syncope. She experienced 8 syncopal episodes over the previous 6 months triggered by laughing, coughing, or emotional distress. Each lasted several minutes without convulsions, auras, or postictal period. In the last year, she had headaches, muscle cramps, constipation, and palpitations but no vomiting or diarrhea. She had a history of chronic hypokalemia of unknown etiology and otherwise no known renal, cardiac, endocrine, or psychiatric diseases. She had at least 2 syncopal episodes during emotional stress as a teenager but did not recur until recently. She drank several liters of water daily, did not have salt cravings, and had previously been advised to increase her dietary K^+^ intake due to hypokalemia. She intermittently took KCl, NaCl, and MgO supplements, though did not take other medications, including laxatives and diuretics. She did not consume tobacco, alcohol, cocaine, or use intravenous drugs. She was born full‐term with no known complications. As a child, she suffered from leg paresthesias while walking and muscle cramps at night along with enuresis until around 12 years of age. Family history was notable for consanguinity between her parents who are first cousins and that her sister also took K^+^ supplements for muscle cramps related to chronic hypokalemia, but none had a history of syncope.

**FIGURE 1:**
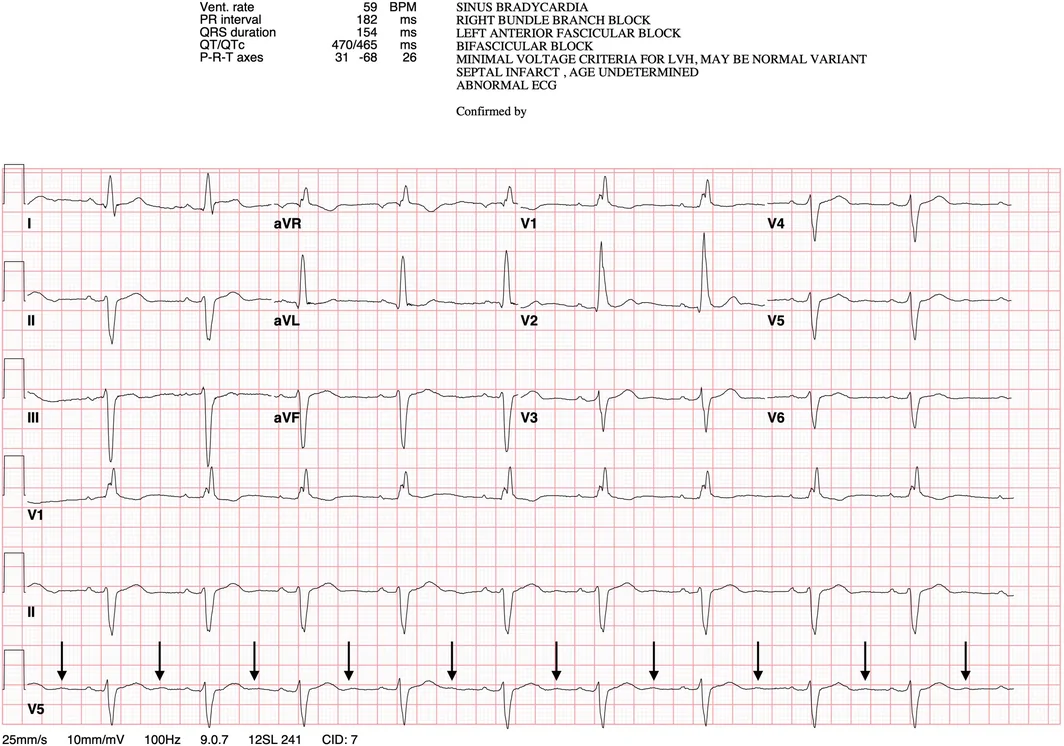
Electrocardiogram with sinus bradycardia, QRS interval 154 ms with right bundle branch and left anterior fascicular blocks, and QTc interval 465 ms with U waves (arrows).

On presentation, she was 5′4″ tall, weighed 84 kg, and her vitals showed a blood pressure of 97/62 mmHg, heart rate of 60 beats/min, normal respiratory rate, and normal peripheral oxygen saturation. Physical exam was within normal limits. Laboratory data revealed hypokalemic metabolic alkalosis with hypomagnesemia, hypocalciuria, and normotensive secondary hyperreninism (Table 1). A diuretic screen was not performed. Electrocardiogram showed sinus bradycardia, left axis deviation, QRS interval 154 ms with right bundle branch and left anterior fascicular blocks, and QTc interval 465 ms with U waves (Figure 1). K^+^ was repleted, no cardiac events were recorded on telemetry, and she was discharged. While taking KCl 60 mEq BID, MgO 420 mg BID, NaCl 1 g daily, and amiloride 5 mg daily, additional testing with continuous cardiac event telemetry monitoring over 2 weeks during 3 syncopal episodes revealed sinus rhythm with occasional premature atrial and ventricular contractions but no arrhythmias. Transthoracic echocardiography, myocardial perfusion imaging, kidney/bladder ultrasonography, electroencephalography, and computed tomography and magnetic resonance imaging of the head and neck were normal. Tilt‐table testing revealed distal sudomotor deficits with borderline increases in heart rate and blood pressure during upright tilt, concerning for early autonomic neuropathy. Given the urinary K^+^ wasting and hypocalciuria, Natera Renasight genetic testing was performed and revealed a homozygous pathogenic missense mutation of c.2221G>A (p.Gly741Arg; rs138977195) of the *SLC12A3* gene encoding NCC, confirming GS.

**TABLE 1:** Relevant blood pressure and serum, plasma, and urine values at different time‐points: Before hospitalization (5 encounters over 2 years), during hospitalization (8 encounters over 5 days), after hospitalization (8 encounters over 5 months), during intensive outpatient treatment (3 encounters over 2 months), and outpatient after intensive treatment (10 encounters over 4 years).

	Reference range	Outpatient before hospitalization	During hospitalization	Outpatient after hospitalization	Outpatient during intensive treatment	Outpatient after intensive treatment
Vital signs—sitting						
SBP (mmHg)	—	118 (102–126)	96 (82–117)	122 (101–137)	126 (115–133)	116 (106–126)
DBP (mmHg)	—	76 (63–84)	58 (50–69)	77 (68–84)	83 (80–85)	76 (66–84)
Heart rate (bpm)	—	90 (77–108)	63 (54–81)	78 (65–90)	62 (56–69)	81 (59–96)
Vital signs—standing						
SBP (mmHg)	—	—	—	116	122 (115–128)	121 (115–126)
DBP (mmHg)	—	—	—	81	82 (80–84)	82 (75–88)
Heart rate (bpm)	—	—	—	70	66 (62–69)	79 (63–95)
Serum						
Na^+^ (mEq/L)	135–145	143 (141–144)	141 (138–144)	143 (140–144)	143 (142–144)	142 (139–144)
K^+^ (mEq/L)	3.5–5.2	2.8 (2.4–3.1)	3.0 (2.5–3.6)	2.6 (2.3–3.0)	3.2 (3.1–3.2)	2.5 (2.1–2.8)
Cl^−^ (mEq/L)	96–108	101 (100–102)	103 (99–107)	101 (98–104)	105 (103–106)	100 (98–104)
HCO_3_^−^ (mEq/L)	22.0–30.0	29.4 (24.3–32.2)	27.6 (26.1–30.2)	28.7 (26.3–34.7)	26.2 (23.9–28.1)	28.3 (25.8–30.2)
Ca^2+^ (mg/dL)	8.5–10.5	9.8 (9.6–10.2)	9.2 (8.8–9.7)	9.6 (8.8–10.0)	9.2 (9.0–9.4)	9.5 (8.3–10.0)
Mg^2+^ (mg/dL)	1.5–2.5	1.6 (1.4–1.7)	1.9 (1.4–2.7)	1.6 (1.4–1.7)	2.0 (1.8–2.1)	1.6 (1.4–1.8)
creatinine (mg/dL)	0.50–1.10	0.86 (0.84–0.88)	0.83 (0.74–0.91)	0.91 (0.85–0.95)	0.91 (0.83–0.99)	0.92 (0.81–0.97)
eGFR (mL/min/1.73m^2^)	—	78 (76–80)	85 (75–97)	76 (72–82)	75 (68–84)	74 (67–85)
Plasma						
K^+^ (mEq/L)	3.5–5.0	2.4	—	2.1 (1.9–2.3)	2.6 (2.5–2.7)	2.0 (1.7–2.2)
Aldosterone (ng/dL)	0.0–30.0	—	9.3	—	11.3 (9.9–12.3)	2.7
Renin activity (ng/mL/h)	0.167–5.380	—	16.686	—	18.493 (13.784–23.253)	12.044
Spot urine						
Na^+^ (mEq/L)	—	69	48 (46–50)	65 (52–95)	138 (102–162)	52 (23–84)
K^+^ (mEq/L)	—	58 (51–64)	49 (41–57)	42 (36–50)	14 (<8–27)^a^	48 (40–52)
Cl^−^ (mEq/L)	—	50	69 (68–69)	67 (52–86)	82 (79–86)	60 (29–87)
Mg^2+^ (mg/dL)	—	—	7.1	<1.4	5.9 (4.9–6.8)	6.8 (6.0–8.3)
Creatinine (mg/dL)	—	97	101 (99–104)	88 (84–92)	95.0 (90.7–99.2)	100 (76–137)
pH (−log[H^+^])	5.0–8.0	7.0 (7.0–7.0)	—	7.5 (7.0–8.0)	7.5 (7.0–8.0)	7.0 (7.0–7.0)
Osmolality (mOsm/kg)	50–1200	—	—	418 (397–444)	452 (449–455)	416
Fractional excretion						
Na^+^ (%)	—	—	0.30 (0.28–0.32)	0.53 (0.35–0.71)	0.87 (0.78–0.95)	0.40 (0.12–0.69)
K^+^ (%)	—	18.5	17.1 (15.0–19.1)	16.2 (13.8–18.6)	5.6 (1.9–9.2)	20.8 (15.5–26.5)
Cl^−^ (%)	—	—	0.61 (0.57–0.64)	0.68 (0.47–0.89)	0.74 (0.65–0.84)	0.65 (0.21–1.04)
Mg^2+^ (%)	—	—	—	0.9	3.0 (2.3–3.7)	4.4 (4.1–4.7)
24‐h urine						
Total volume (mL)	—	—	2400	—	—	—
pH (−log[H^+^])	5.0–8.0	—	7.0	—	—	—
Na^+^ (mEq)	100–210	—	113	—	—	—
K^+^ (mEq)	40–80	—	109	—	—	—
Cl^−^ (mEq)	110–250	—	187	—	—	—
Ca^2+^ (mg)	50–300	—	48	—	—	—
Mg^2+^ (mg)	12–293	—	170	—	—	—

*Note*: Results are presented as mean and (range).

Abbreviations: DBP, diastolic blood pressure, eGFR, estimated glomerular filtration rate; SBP, systolic blood pressure.

^a^ Since 2 spot urine K^+^ values were below the limit of detection (<8 mEq/L), 7 mEq/L was used to calculate the mean.

**FIGURE 2:**
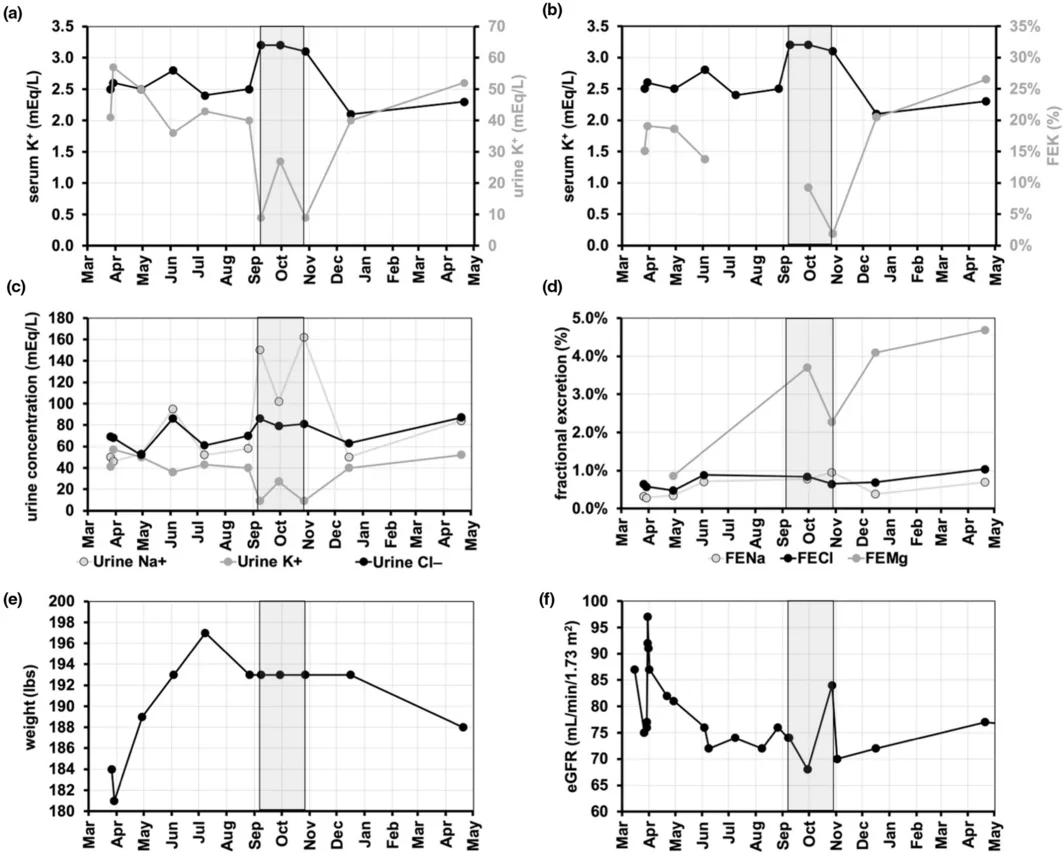
(a) Serum and urine K^+^ concentrations during 1 year of outpatient follow‐up. Hypokalemia improved and kaliuresis was suppressed over a 2‐month period while taking KCl, NaCl, MgO, amiloride, midodrine, and naproxen (gray shading); (b) Serum K^+^ concentrations and Fractional Excretion of K^+^ (FEK); (c) Urine concentration of Na^+^, K^+^, and Cl^−^; d) Fractional excretions of Na^+^ (FENa), Cl^−^ (FECl) and Mg^2+^ (FEMg); (e) weights; and (f) eGFR calculated by CKD‐EPI 2021.

As an outpatient, hypokalemia and hypomagnesemia were refractory to a total daily dose of KCl 240 mEq and MgO 800 mg (Table 1) (Figure 2). To facilitate her safe return to work, amiloride was increased to 10 mg, and due to the possibility of worsening volume contraction with a higher dose diuretic, midodrine 10 mg TID and naproxen 375 mg BID were initiated to raise blood pressure and reduce urine electrolyte excretion. Monthly spot urine and serum K^+^ measurements guided titration of amiloride up to 20 mg daily until kaliuresis was suppressed, hypokalemia improved, and she did not syncopize (Table 1) (Figure 2, shaded area). Titration of NaCl and naproxen was limited by palatability issues and dyspepsia. However, after about 3 months, due to an unexpected major life event, she stopped taking all prescriptions and she was reluctant to resume them except for KCl. Kaliuresis with hypokalemia and syncope recurred (Table 1) (Figure 2).

## 3 DISCUSSION

Recurrent syncope in the setting of chronic hypokalemia ultimately led to a diagnosis of GS. Hypokalemia and hypomagnesemia can cause cardiac conduction delays, manifested by prolonged PR, QRS, and QT intervals (Foglia et al., 2004). Palpitations and cardiac arrhythmias occur in up to 60% of GS patients, and although rare, ventricular tachycardia or sudden cardiac death may also occur (Giordani et al., 2025). Although she had hypokalemia in her teenage years, a diagnosis of GS was not obtained until adulthood when recurrent syncopal episodes that occurred while out‐and‐about posed a threat to her life. Autonomic nervous system dysfunction typically contributes to the pathogenesis of orthostatic hypotension and syncope. However, one study of people with GS found that sympathetic and parasympathetic nervous system function was preserved despite the presence of orthostatic hypotension (Sartori et al., 2007). GS is typically associated with low peripheral resistance and impaired vasoconstriction (Bezzeccheri et al., 2022), which may have contributed to recurrent syncope in our patient who had early signs of autonomic dysfunction. It is possible that syncope was unrelated to hypokalemia but rather was secondary only to volume depletion. In this case, midodrine and/or naproxen without KCl potentially could have helped. Regardless, severe hypokalemia with ongoing renal wasting warranted aggressive treatment to improve muscle cramps and gut motility. Also, hypokalemia in the setting of atrioventricular conduction delays and occasional premature ventricular contractions was still concerning and warranted K^+^ repletion even without an arrhythmia on telemetry monitoring.

The *SLC12A3* c.2221G>A (p.Gly741Arg) mutation identified in our patient was one of the original mutations discovered to cause GS (Simon et al., 1996). It is one of the most common pathogenic missense mutations in *SLC12A3* reported in numerous cohorts (Glaudemans et al., 2012; Li & Gu, 2022; Vargas‐Poussou et al., 2011) with a frequency of 0.04% (104/282254 alleles) in the Broad gnomAD dataset and causes a total or partial loss of protein transporter function when expressed in vitro (De Jong et al., 2002; Kunchaparty et al., 1999).

The hallmark feature of GS is urinary electrolyte wasting. A quantitative clinical approach with measurement of urine electrolytes aids in the evaluation and management of fluid, electrolyte, and acid–base disorders (Kamel & Halperin, 2021; Rein et al., 2020). Urine electrolyte excretion can vary widely and “normal” reference ranges are not established. Thus, spot urine electrolytes should be interpreted relative to the expected renal response for the clinical situation (Kamel & Halperin, 2021; Sterns, 2020; Uribarri et al., 2022). Urine K^+^ excretion normally decreases to 10–15 mEq/d with dietary K^+^ restriction (Kamel & Halperin, 2021). Hence, kaliuresis (>30–40 mEq/d) in the setting of hypokalemia is clearly indicative of renal K^+^ wasting. Additionally, urine water content influences spot urine electrolyte concentrations. As such, measured K^+^ will be lower in dilute urine and higher in concentrated urine. Interestingly, urine osmolality for our patient was remarkably consistent (Table 1), facilitating straightforward comparison of serial urine K^+^ values. Although not formally evaluated but suggestive of a urine concentrating defect, an acquired partial vasopressin resistance from chronic hypokalemia (Al‐Qusairi et al., 2021; Patel et al., 2023) may have contributed to urinary water losses and exacerbated volume depletion.

In fact, vasopressin resistance may occur with GS, at least partially. One study of children and adolescents with GS revealed a significantly lower median urinary osmolality after overnight water deprivation than people without GS (626–699–749 vs. 931–977–1104 mmol/kg; 25th percentile–median–75th percentile) (Peters et al., 1995). While children with GS have a higher mean urine osmolality than people with Bartter syndrome (634 ± 65 vs. 285 ± 8 mOsm/kg) (Walsh et al., 2018), subtle impairments in water homeostasis may be underrecognized and underappreciated. Whether urine concentrating defects in GS are due to hypokalemia or other mechanisms related to impaired NCC function remains to be determined.

Chronic hypokalemia or metabolic alkalosis, characteristics of GS, should prompt investigation into the etiology. Several pathophysiological mechanisms of GS contribute to kaliuresis and hypokalemic metabolic alkalosis. Reduced NCC transporter activity in the DCT allows for more distal NaCl delivery to the collecting duct (CD), which activates electrogenic Na^+^ absorption via epithelial sodium channels (ENaC) and K^+^ secretion via renal outer medullary potassium channels (ROMK) and Maxi‐K (BK) channels (Lasaad et al., 2024; Simon et al., 1996). High tubular flow in the CD causes flow‐induced K^+^ secretion by BK channels as well as increases prostaglandin E_2_ (PGE_2_) synthesis (Flores et al., 2012) which also stimulates K^+^ secretion. A role for PGE_2_ is established for Bartter syndrome but remains unclear for GS (Blanchard et al., 2017). Additionally, secondary hyperaldosteronism from mild volume depletion further stimulates tubular K^+^ secretion in the CD. However, hypokalemia and K^+^ depletion may attenuate aldosterone secretion, independent of changes in sodium balance or intravascular volume (Gann et al., 1964), and mask overt hyperaldosteronism secondary to salt wasting and volume depletion characteristic of GS.

Accordingly, treatment of GS involves lifelong high oral intake of Na^+^, K^+^, Cl^−^, and Mg^2+^ salts in food and tablets (Blanchard et al., 2017). In some cases, K^+^‐sparing diuretics, nonsteroidal anti‐inflammatory drugs (NSAIDs), and renin‐angiotensin‐aldosterone system antagonists may also be prescribed. NSAIDs can reduce renin secretion during hypovolemia (Schlondorff, 1993). Mineralocorticoid receptor antagonists and ENaC inhibitors improve hypokalemia and kaliuresis in patients with GS by combating aldosterone and flow‐induced K^+^ secretion in the ASDN. However, diuretics also decrease extracellular fluid volume, which may exacerbate orthostatic hypotension, although this decrease had no obvious clinical impact in one study of six patients with GS (Colussi et al., 1994). As a rare disease, tolerability, efficacy, and safety data for GS treatments are limited. A randomized crossover study of 30 people with GS taking K^+^ and Mg^2+^ supplements demonstrated that indomethacin 75 mg, eplerenone 150 mg, and amiloride 20 mg can each increase plasma K^+^ concentration (Blanchard et al., 2015). Indomethacin was most effective but caused more gastrointestinal symptoms and reduced kidney function.

Serial spot urine K^+^ measurements were important to estimate the severity of urinary K^+^ wasting. Although one could argue that serum K^+^ alone is sufficient to guide medication dose titration, hypokalemia may be resistant to treatment, and quantification of urinary K^+^ wasting can direct how much oral K^+^ repletion is needed to achieve net positive balance. Additionally, spot urine electrolytes are easier to obtain than 24‐h urine collections and provide valuable information when interpreted simultaneously with serum electrolytes. With K^+^ sparing diuretics, urine K^+^ measurements inform when kaliuresis is suppressed, and at that time, additional treatment interventions that reduce kaliuresis would be of limited value while KCl supplementation would be more efficacious. We unfortunately did not have sufficient time while kaliuresis was suppressed to determine if normal serum K^+^ levels could be achieved with higher KCl dosing. Additionally, suppression of kaliuresis was an objective measure that she was adherent to taking new medications. KCl alone would not have reduced urine K^+^ excretion. Ultimately, knowledge gaps remain and research is needed to determine the urine values and creatinine ratios that establish wasting of K^+^, Na^+^, and Cl^−^ (Blanchard et al., 2017).

Here, we present a unique and logical approach for treating GS‐associated hypokalemia and recurrent syncope that was guided by serial urine electrolyte measurements. Urine electrolytes should be integrated into routine clinical practice for patients with blood pressure or electrolyte disorders. Genetic testing for renal electrolyte transport‐related gene mutations among patients with recurrent syncope and chronic electrolyte abnormalities may be diagnostic.

## AUTHOR CONTRIBUTIONS

**Saloni Agrawal:** Conceptualization; data curation. **Kenneth M. Fifer:** Conceptualization; data curation; investigation. **Samira S. Farouk:** Conceptualization; supervision. **Joshua L. Rein:** Conceptualization; data curation; formal analysis; investigation; supervision.

## CONFLICT OF INTEREST STATEMENT

None.

## ETHICS STATEMENT

Written publication consent was obtained from the patient. Approval from the Institutional Review Board of the Icahn School of Medicine at Mount Sinai was not required for a single case report. Additional clinical data and non‐protected health information may be available upon reasonable request.

## DISCLAIMER

The views expressed in this article are those of the authors and do not necessarily reflect the position or policy of the Department of Veterans Affairs or the United States government.

## Supporting information


**Data S1.** CARE Checklist of information to include when writing a case report.
