# Supplementary material for: Urine electrolytes guide treatment of Gitelman syndrome and recurrent syncope—A case report
Source: Physiol Rep. 2026 Sep 21;14(18):e71043. doi: 10.14814/phy2.71043 (PMC13591546; doi:10.14814/phy2.71043)
Supplement: Supplementary file 1 [file PHY2-14-e71043-s001.pdf]

CARE Checklist of information to include when writing a case report

CARE case report guidelines

(cc) BY-NC-ND

Topic	Item	Checklist item description	Reported on Line
Title	1	The diagnosis or intervention of primary focus followed by the words "case report"	1
Key Words	2	2 to 5 key words that identify diagnoses or interventions in this case report, including "case report"	34
Abstract (no references)	3a	Introduction: What is unique about this case and what does it add to the scientific literature?	63-66
	3b	Main symptoms and/or important clinical findings	70-91
	3c	The main diagnoses, therapeutic interventions, and outcomes	89-100
	3d	Conclusion—What is the main "take-away" lesson(s) from this case?	191-195
Introduction	4	One or two paragraphs summarizing why this case is unique (**may include references**)	101-152
Patient Information	5a	De-identified patient specific information.	70-91
	5b	Primary concerns and symptoms of the patient.	70-74
	5c	Medical, family, and psycho-social history including relevant genetic information	70-83
	5d	Relevant past interventions with outcomes	70-79
Clinical Findings	6	Describe significant physical examination (PE) and important clinical findings.	85-100
Timeline	7	Historical and current information from this episode of care organized as a timeline	70-98
Diagnostic Assessment	8a	Diagnostic testing (such as PE, laboratory testing, imaging, surveys).	87-100
	8b	Diagnostic challenges (such as access to testing, financial, or cultural)	n/a
	8c	Diagnosis (including other diagnoses considered)	93-94, 96-100
	8d	Prognosis (such as staging in oncology) where applicable	102-110
Therapeutic Intervention	9a	Types of therapeutic intervention (such as pharmacologic, surgical, preventive, self-care)	90-110
	9b	Administration of therapeutic intervention (such as dosage, strength, duration)	90-110
	9c	Changes in therapeutic intervention (with rationale)	90-110
Follow-up and Outcomes	10a	Clinician and patient-assessed outcomes (if available)	90-110
	10b	Important follow-up diagnostic and other test results	93-98
	10c	Intervention adherence and tolerability (How was this assessed?)	102-110
	10d	Adverse and unanticipated events	108-110
Discussion	11a	A scientific discussion of the strengths AND limitations associated with this case report	113-152, 178-189
	11b	Discussion of the relevant medical literature **with references**.	113-189
	11c	The scientific rationale for any conclusions (including assessment of possible causes)	113-189
	11d	The primary "take-away" lessons of this case report (without references) in a one paragraph conclusion	191-195
Patient Perspective	12	The patient should share their perspective in one to two paragraphs on the treatment(s) they received	n/a
Informed Consent	13	Did the patient give informed consent? Please provide if requested	**Yes** ☒ **No** ☐
